# Wide-Field High-Speed Scanning Acoustic/Photoacoustic Microscopy for Whole-Body Imaging of Small Animals

**DOI:** 10.3390/bios15040200

**Published:** 2025-03-21

**Authors:** Joongho Ahn, Hyoseok Choi, Seongjun Lim, Jin Young Kim, Jeongwoo Park

**Affiliations:** 1 Departments of Electrical Engineering and Convergence IT Engineering, Medical Device Innovation Center, Pohang University of Science and Technology, Pohang 37673, Republic of Korea; joongho.ahn@postech.ac.kr (J.A.); hyoseokchoi@postech.ac.kr (H.C.); 2 Opticho Inc., Pohang 37673, Republic of Korea; 3 Department of Biomedical Convergence Science and Technology, Kyungpook National University, Daegu 41566, Republic of Korea; sjgj0624@knu.ac.kr; 4 Department of Advanced Bioconvergence, Kyungpook National University, Daegu 41566, Republic of Korea; 5 Cell and Matrix Research Institute, Kyungpook National University, Daegu 41944, Republic of Korea

**Keywords:** multimodal imaging, preclinical imaging, photoacoustic imaging, ultrasound imaging, transparent ultrasound transducer

## Abstract

Photoacoustic (PA) imaging combines optical contrast with ultrasound (US) detection, enabling high-resolution imaging of biological tissues with greater penetration depth than conventional optical techniques. Among its various implementations, photoacoustic microscopy (PAM) achieves micrometer-scale resolution by focusing laser excitation and detecting ultrasonic signals, allowing for the detailed visualization of microvascular structures and fine tissue morphology. Over the last decade, PAM imaging speed has significantly increased by adopting PA scanners that steer optical and acoustic waves. However, these scanners must be placed after focusing optics to co-align the waves on a spot, which creates bent focal lines along the scanning direction and limits the scanning range. To achieve wide-field imaging, various image mosaic algorithms have been applied, but these methods require multiple manual operations, which take more time than the imaging itself. In this study, we developed a wide-field, high-speed scanning acoustic/photoacoustic microscopy (SA/PAM) system equipped with a transparent ultrasound transducer and a moving magnet linear stage, which eliminates the need for complex mosaic algorithms. This system enables wide-field imaging up to 50 × 50 mm^2^ while maintaining high lateral resolution, achieving an imaging speed of 50 Hz in a B-scan image. Through in vivo mouse US/PA imaging, the system demonstrated its capability to visualize blood vessels and organs across the whole body of small animals. These findings suggest that the SA/PAM system is a practical tool for biomedical research, allowing for efficient visualization of vascular networks and anatomical structures in various preclinical studies.

## 1. Introduction

Photoacoustic (PA) imaging, a hybrid imaging technique that combines optical and acoustic methodologies, has significantly advanced over the past several decades [1,2,3,4,5,6,7]. PA imaging is based on the PA effect, where ultrasound (US) waves are generated when a material absorbs pulsed light, converting optical energy into heat and inducing thermoelastic expansion. This technique has found extensive applications in biomedical research due to its ability to leverage endogenous chromophores in biological tissues, such as melanin, cell nuclei, hemoglobin, and lipids [8,9]. By exploiting the directional properties of light and US waves, PA imaging achieves multiscale imaging capabilities, facilitating the development of diverse imaging platforms [10,11]. By focusing on a specific point and achieving high resolution, it can be used as a microscope, known as PA microscopy (PAM) [11,12,13,14,15,16,17]. PAM has been widely employed in biomedical studies such as digital histology [18,19,20,21,22,23,24], vascular response analysis [25,26,27,28,29,30,31,32,33], and drug delivery monitoring [34,35,36,37,38,39,40], offering unique optical absorption-based contrast, as opposed to the reflection or scattering contrasts provided by traditional optical microscopy. However, technical hurdles in PAM implementation remain, as the light and US waves must be aligned, and PA signals should be obtained point by point for each aligned position.

To address the hurdles and achieve the improvements in PAM, versatile modules for PA imaging, such as beam combiners [41,42,43] and scanners [44,45,46], have been continuously developed. First, the beam combiner is an essential component to co-align light and US waves, obtaining a high signal-to-noise ratio (SNR). The first co-aligning module that escaped from the off-axis alignment of light and US waves was an opto-US beam combiner. The light and US waves entering the other input ports of the beam combiner reflect or go straight, respectively, and coaxially come out to one output port with co-aligned. However, optical and acoustic losses occur whenever they pass subparts of the beam combiner [12]. The second approach is a ring-shaped US transducer (RUT) that has the hole in the center [47,48]. The light passes through the hole without any interferences, so there is no optical loss. However, due to the constraint of putting light into the hole, limited optical beam width and optical numerical aperture (NA) exist, negatively affecting the resolution. The decrease in the RUT’s sensitivity is also inevitable because its active area receiving US waves is reduced as much as the center hole’s size [9]. As a better alternative, a transparent US transducer (TUT) has been developed [49,50,51,52,53,54,55]. Because the TUT is optically transparent and ultrasonically active in its entire area, the light can pass, and the US waves can be received with little optical and acoustic losses.

In addition to beam combiners, scanners are the other key component to concurrently steer light and US waves for high-speed imaging. The first-developed high-speed PA scanner was an immersible micro-electro-mechanical systems (MEMS) scanner and achieved cross-sectional B-mode imaging speed up to 400 Hz [56]. Subsequently, a galvanometer scanner was utilized with only a mirror immersed in water. It increased the B-mode imaging speed up to 500 Hz [57]. Using a galvanometer scanner, a scanning speed of 100 Hz over a 1 mm range was achieved [58]. Additionally, with spiral scanning, image acquisition over a 12.5 cm^2^ area was achieved in 6.4 s [59,60]. Furthermore, a polygon-mirror scanner on which the six mirrors were attached on the sixth side of the polygon structure improved the B-mode imaging speed up to 900 Hz [61]. More recently, a 12-facet polygon scanner achieved a volumetric imaging rate of 2 Hz over 11 × 7.5 × 1.5 mm^3^ [33].

Although these scanners achieved high-speed PA imaging capability in PAMs, the scanners should be at the end after focusing and co-aligning light and US waves. In the configuration where the scanner is placed at the end, the farther the scanline is from the scanning axis, the shorter the focal length. This causes the edges of the scan range to be out of focus when scanning flat samples, limiting the scannable range. Moreover, traditional approaches to wide-field imaging in PAM have often relied on image stitching algorithms to expand the field of view (FOV) for the whole-body imaging of small animals. However, these methods introduce spatial distortions and require manual adjustments, increasing image acquisition and processing time. To address these challenges, different correction algorithms such as scanline linearization, coordinate conversion, and seamless image mosaic were developed, leading to the release of 3D Photoacoustic Visualization Studio (3D PHOVIS) [62]. Nevertheless, these algorithms still require extensive manual intervention, leading to significant variability in image quality depending on the user’s background knowledge, experience, and expertise. As a result, the post-processing steps often take longer than the actual image acquisition, increasing the total time from scanning to final image display.

Those issues can be resolved by employing a fast linear stage with a moving magnet [63]. The moving-magnet-based linear stages (MLSs) are based on magnetic drive and have less friction and backlash compared to step-motor-based linear stages (SLSs). With little friction, the MLSs (~1000 mm/s) are much faster than SLSs (~20 mm/s). The imaging mechanism returns to mechanical scanning of the imaging head, including optical components and a single-element US transducer, so it takes back the advantages scarified by the use of the scanners. Even when scanning a long distance, the imaging head moves directly, so the geometric issues associated with the scanners do not occur, and no algorithms to calibrate them are needed. In addition, the PA imaging can be performed simultaneously with ultrasound imaging because the scanning range is much wider than the US beam width.

In this study, we developed wide-field, high-speed scanning acoustic/photoacoustic microscopy (SA/PAM) using TUT and MLS. For characterizing the new SA/PAM system, the phantoms of a carbon fiber and a leaf skeleton were imaged. Further, in vivo US/PA imaging of a mouse was performed and the blood vessels and organ in the mouse’s whole body were intuitively visualized without much effort. From these results, we believe that wide-field, high-speed SA/PAM could be used in various preclinical studies for whole-body imaging applications such as drug delivery monitoring.

## 2. Materials and Methods

### 2.1. Implementation of Wide-Field, High-Speed Photoacoustic Microscopy

**Figure 1:**
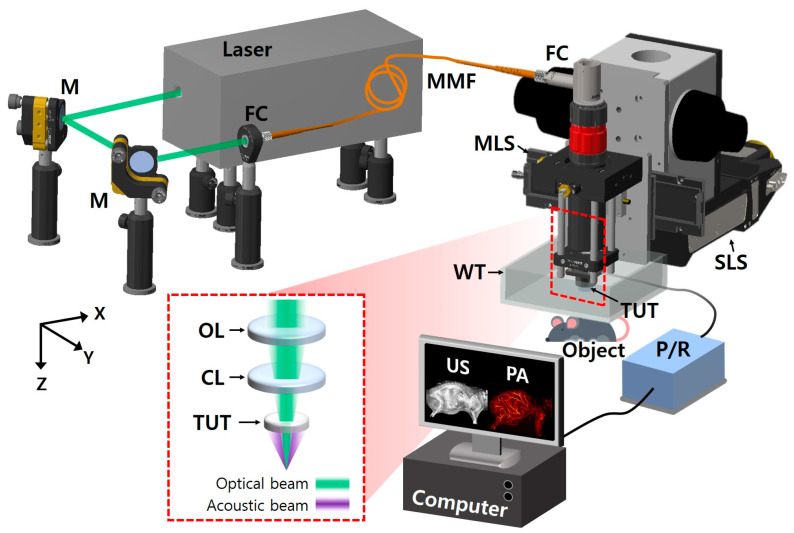
Schematic of wide-field and high-speed SA/PAM system. M, mirror; FC, fiber collimator; MMF, multimode fiber; MLS, moving-magnet-based linear stage; SLS, step-motor-based linear stage; TUT, transparent ultrasound transducer; US, ultrasound; PA, photoacoustic, P/R, pulser receiver; WT, water tank; OL, objective lens; and CL, correction lens.

Figure 1 shows the graphical representation of the developed SA/PAM system, which has optical source and imaging subsystems. For PA imaging, the nanosecond pulsed light from a 532 nm pulsed laser (Sol 5W, Bright Solutions, Prado, Italy) was steered using two flat mirrors (M, BB1-E02, Thorlabs, NJ, USA) and coupled to a multimode optical fiber with 105 µm core (MMF, M43L01, Thorlabs, Newton, NJ, USA) through a fiber-optic coupler (TC12FC-543, Thorlabs, Newton, NJ, USA) in the optical source subsystem. By connecting the other end of the optical fiber to a fiber collimator (FC, RC08FC-P01, Thorlabs, Newton, NJ, USA) in the SA/PAM’s imaging subsystem, the light from the optical source subsystem was delivered to the imaging subsystem. The collimated light from the fiber collimator was focused on targets using a focusing achromatic doublet lens (AC254-050-A, Thorlabs, Newton, NJ, USA). Between the focusing lens and targets, the light being focused passed through a customized TUT, which has the following specifications: 9 mm diameter, 20 mm focal length, 13 MHz center frequency, 60% fractional bandwidth, and 75% optical transmittance at 532 nm. To compensate for the optical path diverged by the concave shape of the acoustic lens in the transducer, a correction lens (AC254-100-A, Thorlabs, Newton, NJ, USA) was inserted between the fiber collimator and focusing lens. Through the optical components and TUT, light illuminated targets, where the US waves generated by the PA effect returned to the TUT, converted to PA signals, and finally amplified with 40 dB gain by a US pulser receiver (5072PR, Olympus NDT, Waltham, MA, USA). For US imaging, the US pulser receiver delivered high-voltage impulses to the TUT, and the high-voltage electrical signals were converted to US waves. The excited US waves were reflected at the boundaries of the targets and the reflected US echoes returned to the TUT. The US echoes were converted to US signals and amplified by the TUT and US pulser receiver, respectively.

The imaging head with the collimator, lenses, transducer, and amplifier was fixed on an MLS (NT55V65, IKO International, Tokyo, Japan) and a SLS (L-509.20SD00, Physik Instrumente, Karlsruhe, Germany) for high-speed scanning with a long travel range along X axis and an extend FOV along Y axis, respectively. Meanwhile, the amplified US/PA signals were converted to digital signals by a digitizer card (ATS9350, Alazar technologies, Pointe-Claire, QC, Canada) with a 250 MS/s sampling rate and transferred to a computer. The operations of the pulsed laser, US pulser receiver, two linear stages, and digitizer card were synchronized by the pre-defined sequences in a multifunction I/O device (PCIe-6321, National Instruments, Austin, TX, USA) and LabVIEW (National Instruments, Austin, TX, USA) for simultaneous US/PA imaging.

### 2.2. Phantom Prepration and Imaging Method

For identifying the SA/PAM system’s characteristics, a carbon fiber of about 7 µm was prepared to measure US/PA imaging resolutions. Cross-sectional B-mode imaging of the carbon fiber with a 5 µm interval was conducted. From the images of the carbon fiber, the resolutions were measured using the full widths at half maximum (FWHMs) methodology. In addition, to confirm wide-field and high-speed US/PA imaging capability, a leaf skeleton was attached to stacked sheets of slide glass using an Ethyl cyanoacrylate adhesive glue (Loctite 401, Henkel, Düsseldorf, Germany). To fully visualize the leaf skeleton, an area of 50 × 30 × 37.5 mm^3^ was imaged with 20, 25, and 24 µm intervals along X, Y, and Z axes, respectively. Then, the image processing and visualization were performed in MATLAB 2018b (MathWorks, Natick, MA, USA) and 3D PHOVIS. In 3D PHOVIS, PA and US data were accumulated from 1200 B-scan frames to reconstruct three-dimensional PA and US volumetric images. To generate images for visualization, maximum amplitude projection (MAP) and maximum intensity projection (MIP) algorithm were applied along the Z axis for both PA and US volumetric data, respectively. Finally, these PA and US images were overlaid to create a co-registered PA MAP/US MIP image.

### 2.3. Animal Preparation

The animal experiment followed the laboratory animal protocol approved by the Institutional Animal Care and Use Committee (IACUC) of Pohang University of Science and Technology. To validate the SA/PAM system’s in vivo imaging capability, two healthy Balb/c nude mice (female, 6 weeks, ~20 g) were prepared. One mouse was used for US/PA imaging of its whole body in supine and lateral positions.

Under the approved experimental protocol, the mouse was anesthetized using 0.5 L/min O2 and 1.5% isoflurane with an anesthesia system (VIP 3000 Veterinary Vaporizer, Midmark, OH, USA). The downy hair on the mouse was removed using a depilatory (Nair hair remover lotion, Church & Dwight, Ewing, NJ, USA), and the mouse, positioned in supine or lateral orientations, was placed on a heating pad installed on the Z-axis manual stage (L490, Thorlabs, Newton, NJ, USA). Next, the mouse was covered with US gel (Ecosonic, Sanipia, Gimje, Republic of Korea) and placed under a thin membrane-sealed water tank for US-wave transmission. Preparation for US/PA imaging was completed by filling the water tank and submerging the imaging head. Finally, simultaneous US/PA imaging was performed on the mouse’s whole body, except its head. After the experiment, the measured optical fluence was 10 mJ/cm^2^, which is below the maximum permissible exposure in skin (20 mJ/cm^2^), as stated by American National Standards Institute. Afterward, the post-processing and graphical representation were also performed in MATLAB and 3D PHOVIS.

## 3. Results

### 3.1. In Vitro Phantom US/PA Imaging

**Figure 2:**
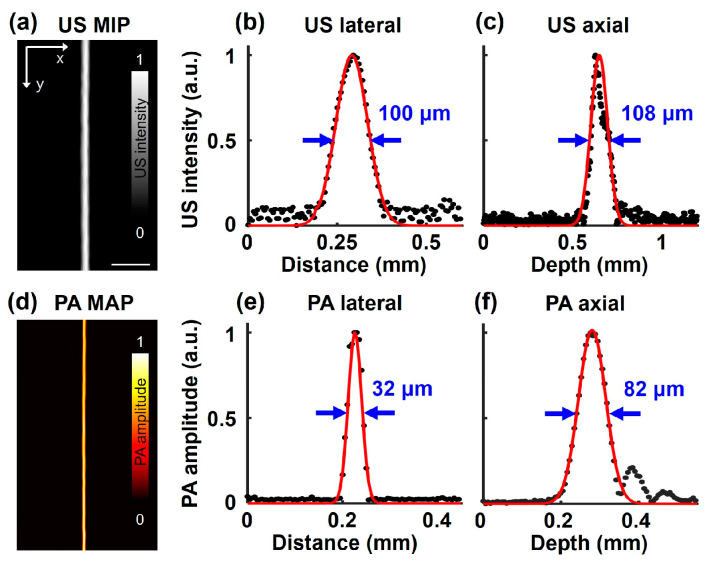
The spatial resolution of SA/PAM system. (**a**) US MIP image of single carbon fiber. (**b**) US lateral and (**c**) axial resolutions, which correspond to 100 and 108 µm, respectively. (**d**) PA MAP image of the caber fiber. (**e**) PA lateral and (**f**) axial resolution, which correspond to 32 and 82 µm, respectively.

To measure the spatial resolutions of the SA/PAM system, three-dimensional US/PA images of the single carbon fiber were obtained. Then, raw data on the horizontal and vertical lines at the carbon fiber were extracted, and line spread functions (LSFs) were derived by approximating the data to a Gaussian formula. Finally, the lateral and axial resolutions of the SA/PAM system were represented as the full width half maxima (FWHMs) of the LSFs. The US MIP image of the carbon fiber is shown in Figure 2a. The measured lateral and axial resolutions from the US pulser are 100 and 108 µm, respectively (Figure 2b,c). The lateral resolution is relatively high due to the high numerical aperture of the TUT, while the axial resolution remains within a reasonable range compared to existing US imaging techniques [64]. A PA MAP image of the carbon fiber is shown in Figure 2d. The measured lateral and axial resolutions from the pulsed laser are 32 and 82 µm, respectively (Figure 2e,f).

**Figure 3:**
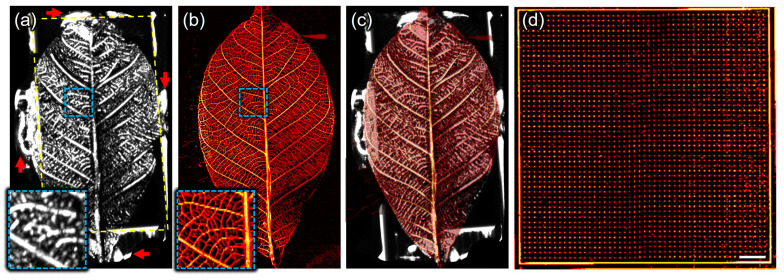
Scanning acoustic and photoacoustic imaging of a leaf skeleton. (**a**) Ultrasound maximum-intensity-projected (US MIP) image, (**b**) photoacoustic maximum-amplitude-projected (PA MAP) image, and (**c**) overlaid image of (**a**,**b**). The outside of yellow-dotted rectangular indicates stacked slide glasses and the red arrows does the adhesive that used to attach the leaf skeleton to the stacked slide glasses. (**d**) PA MAP image of a 50 × 50 mm^2^ wide area dot phantom. The spacing between dots is 200 μm. Scale bar is 5 mm.

Another phantom of a leaf skeleton was used to verify the wide-field US/PA imaging capability of the SA/PAM system. From US/PA imaging of the leaf skeleton attached to the stacked slide glass, volumetric US/PA data were obtained and represented as US MIP/PA MAP images with an FOV of 50 × 30 mm^2^ (Figure 3a,b). The US signals in Figure 3a indicate the echoes on the leaf skeleton (inside a yellow dotted rectangle), stacked slide glasses (outside), and adhesive (red arrows), because those materials have different mechanical impedance and reflect acoustic waves well. On the other hand, the PA signals in Figure 3b represent the laser-induced PA generation on the leaf skeleton only, as the glass and adhesive do not absorb optical fluence. In the magnified image outlined by the blue dotted box, the PA lateral resolution is superior to the US lateral resolution, allowing finer branches of the leaf skeleton to be observed more clearly in the PA MAP image than in the US MIP image. To further analyze the structural correlation between two modalities, the US MIP and PA MAP images were overlaid (Figure 3c), and, finally, the overlaid US MIP and PA MAP images were seamlessly matched. Figure 3d shows the PA MAP image obtained from a 50 × 50 mm^2^ wide-area dot phantom, emphasizing the system’s capability to efficiently image large areas with a single scan. Prior to in vivo experiments, imaging the three kinds of phantoms demonstrated that the developed SA/PAM system is capable of US/PA imaging the whole body of small animals in terms of resolution and FOV.

### 3.2. In Vivo Mouse Whole-Body US/PA Imaging

**Figure 4:**
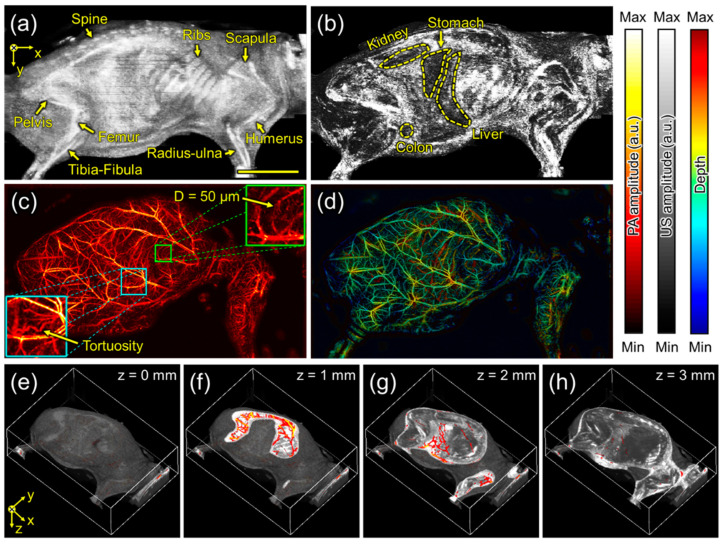
Scanning acoustic and photoacoustic imaging of the mouse in a lateral (lying side) position. (**a**) Ultrasound maximum-intensity-projected (US MIP) image and (**b**) skin-removed US MIP image. (**c**) Photoacoustic maximum-amplitude-projected (PA MAP) image and (**d**) depth-encoded PA MAP image. (**e**–**h**) The overlaid US and PA images in the highlighted depths of 0, 1, 2, and 3 mm. Scale bar is 1 cm.

The US/PA imaging experiment was conducted with the mouse in a lateral (lying side) position. Figure 4a shows the US MIP, where the body shape and bones, including the spine, ribs, scapula, pelvis, femur, tibia-fibula, humerus, and radius-ulna, are observed due to strong US reflection caused by the acoustic impedance differences between water, skin, and bony or other tissues. Figure 4b represents the skin-removed US MIP. To remove the skin layer, it is assumed that the first strong signals acquired in the time domain correspond to the strong reflections caused by the acoustic impedance difference between water (outside the body) and skin (inside). Here, the skin-removed US MIP is extracted by removing pixels with these strong signals from the 2D cross-sectional data and projecting the maximum intensity, which reveals the shapes of organs, including the kidney, stomach, liver, and colon. Meanwhile, Figure 4c shows the PA MAP in the same FOV as the US MIP in Figure 4a, where the blood vessels and their networks are distinctly visualized. Figure 4d presents the depth-encoded PA MAP by replacing the maximum amplitude at each pixel with the depth at which the maximum amplitude is generated. Furthermore, Figure 4e shows a 3D-rendered US image with reduced intensity, and Figure 4f–h show overlay cross-sectional US and PA images measured at 1, 2, and 3 mm relative to the highest detected skin surface in the 3D reconstruction. The 3D overlaid PA/US images combined structural and vascular information, providing a comprehensive view of tissue morphology and microvascular networks.

**Figure 5:**
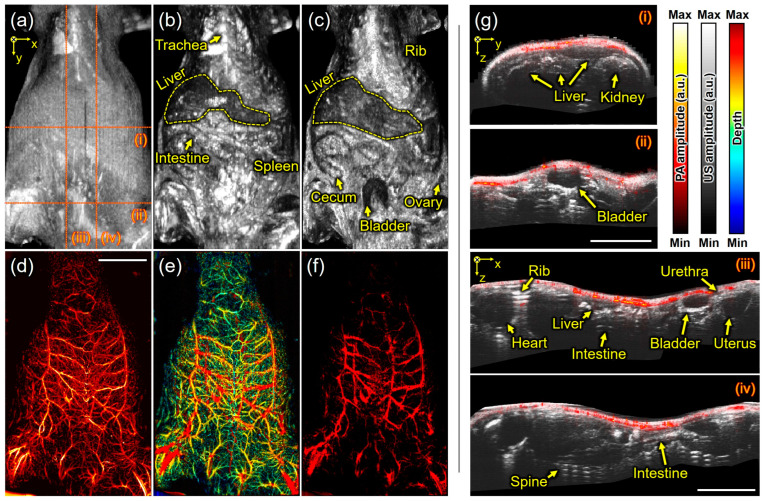
Scanning acoustic and photoacoustic imaging of the mouse in a supine (lying down) position. (**a**) Ultrasound maximum-intensity-projected (US MIP) image, (**b**) skin-removed US MIP image, and (**c**) US MIP image deeper than 1 mm from the skin. (**d**) Photoacoustic maximum-amplitude-projected (PA MAP) image, (**e**) depth-encoded PA MAP image, and (**f**) PA MAP image deeper than 1 mm from the skin. (**g**) The overlaid US and PA images (**i**–**iv**) in the red-dotted lines on (**a**). (**g**) Scale bar is 1 cm.

The other US/PA imaging of the mouse in a supine (lying down) position was performed. Figure 5a represents the US MIP, where the shape of the skin is well observed, but bones are not distinctly visible in this position. Figure 5b shows the skin-removed US MIP image, revealing structures, such as the trachea, liver, intestine, and spleen. Figure 5c, which is the US MIP image after removing US signals from the skin to 1 mm under the skin in the depth direction, reveals a wider area, including the liver, rib, cecum, bladder, and ovary. Meanwhile, Figure 5d,e show the PA MAP and its depth-encoded PA MAP images, respectively. When PA signals shallower than 1 mm from the skin were removed using the same method as in Figure 5c, deeper blood vessels were exposed, as shown in Figure 5f. In more detail, Figure 5g(i–iv) represent the overlaid US and PA cross-sectional images along the red dotted lines in Figure 5a. The upper side of the kidney, which was previously invisible, is now revealed in Figure 5g(i), and the entire bladder can be observed in Figure 5g(ii). In Figure 5g(iii,iv), organs, such as the heart, intestine, uterus, and urethra, can be inferred from the shape and location of tissues, while bones such as the rib and spine (~13 mm) are identified from strong reverberations. Unfortunately, it is difficult to obtain new information from PA images because the expressible depth of PA images is relatively shallower than that of US images, and blood vessels can be found as just small circles in one cross-sectional PA image.

## 4. Discussion

The main research direction in the field of photoacoustic microscopy has been the in vivo observation of small but rapid movements in animals and humans. Accordingly, researchers in this field have focused more on improving imaging speed than other technical factors. They have achieved fast imaging speeds over 50 Hz (considered real time) by oscillating small mirrors and steering optical and acoustic waves, rather than moving large parts directly. However, high-speed mirror-scanning photoacoustic microscopes suffer from geometric drawbacks in areas far from the mirror, causing distortion at the edges of PA images and limiting the FOV. The wide-field high-speed SA/PAM developed in this study overcame these challenges by incorporating a high-speed MLS, despite its low load capacity. This was achieved by reducing the weight of the imaging head using minimal parts, aided by the TUT. In the imaging experiment, our developed system’s imaging speed along the horizontal axis was 2 Hz per 50 mm, which corresponds to 50 Hz per 2 mm in the system with optical steering. Furthermore, the repetitive line scanning, known as raster scanning, maintained lateral resolution without requiring additional algorithms to convert coordinate systems. In contrast, optical steering systems with bent focal lines require polar-to-Cartesian coordinate conversion to compensate for resolution and accurately represent pixels at the same depth along a line. In other words, the developed high-speed PAM system is more efficient for wide-field imaging than the scanner-based high-speed PAM system. It is unfortunate that the developed system could not utilize the maximum speed of the MLS in the experiment because the fast-moving imaging head made waves in the water. It is expected that imaging speed could be increased by optimizing parameters such as weight and acceleration.

In accordance with the capability of long-distance imaging, US imaging becomes meaningful. Scanner-based PAM systems are limited to a line scan range of only a few millimeters and the US lateral resolution of a hundreds of micrometers, resulting in blurred US images only. However, the wide-field US imaging up to 50 mm achieved in this study demonstrated its ability to produce anatomical images, including organs, bones, and other structures. The anatomical information from US imaging enhances the productivity of the PAM system by effectively representing both anatomy and vasculature, even in overlapping views if necessary. With these advantages, the high-speed SA/PAM has demonstrated its practicality in visualizing anatomy and vasculature throughout the entire body of small animals. Unlike scanner-based high-speed imaging, which observes rapid changes in organs, the stage-based high-speed imaging introduced in this study is effective for monitoring gradual changes across the entire body. For example, when tumor cells are injected into mice, they accumulate in lymph nodes distributed throughout the body, and their time-dependent changes can be monitored using the developed SA/PAM system.

Furthermore, while this study primarily focused on small animal imaging, the system’s wide-field scanning capability and adaptable imaging head suggest its potential for use in larger animals. By adjusting the scanning parameters and optical/acoustic configurations, it could also be optimized for imaging medium-sized animals such as rabbits or even larger specimens in ex vivo studies. Building on these capabilities, the state-of-the-art features in US and PA research could be adopted in the developed SA/PAM. First, the current SA/PAM system uses a single optical source with a wavelength of 532 nm. However, other studies focusing on optical sources have employed multiple-wavelength optical sources with advanced techniques such as optical parametric oscillators [65,66,67] and stimulated Raman scattering [68,69] to distinguish materials with different optical absorption coefficients based on their wavelengths. Especially in vascular imaging studies, the spectral unmixing algorithms estimating the components of a mixture using spectral signals has already been applied to distinguish oxy- and deoxy-hemoglobin and calculate hemoglobin oxygen saturation [25,70,71,72]. By applying the multi-wavelength approach to our SA/PAM system, not only blood vessels but also their characteristics could be represented. Second, other optical imaging could be added using the optical transparency of the TUT. Previous studies have demonstrated optical coherence tomography and fluorescence imaging via the TUT, along with multimodal imaging capabilities without hardware changes [49,55]. Since the SA/PAM system’s imaging head includes optical components for focusing on targets, multimodal imaging can be established by simply adding a back-end system for optical imaging.

## 5. Conclusions

In this study, a wide-field, high-speed SA/PAM system was developed and validated through phantom and in vivo experiments. Equipped with the state-of-the-art TUT, the system seamlessly integrates scanning acoustic microscopy (SAM) and PAM, ultimately forming a unified system with dual-imaging capabilities. Additionally, the lightweight imaging head, benefiting from the TUT, enables the system to use a magnet-driven motorized stage with minimal friction, making it faster and more practical than scanner-based PAM for a wide FOV. Based on these advantages, the developed system has demonstrated its capability to image the whole body of small animals and could serve as a practical tool for biomedical research and preclinical studies.

## Data Availability

The raw data supporting the conclusions of this article will be made available by the authors on request.

## References

[1] Xu M., Wang L.V. (2006). Photoacoustic imaging in biomedicine. Rev. Sci. Instrum..

[2] Beard P. (2011). Biomedical photoacoustic imaging. Interface Focus.

[3] Mallidi S., Luke G.P., Emelianov S. (2011). Photoacoustic imaging in cancer detection, diagnosis, and treatment guidance. Trends Biotechnol..

[4] Weber J., Beard P.C., Bohndiek S.E. (2016). Contrast agents for molecular photoacoustic imaging. Nat. Methods.

[5] Wang L. (2017). Photoacoustic Imaging and Spectroscopy.

[6] Choi W., Park B., Choi S., Oh D., Kim J., Kim C. (2023). Recent advances in contrast-enhanced photoacoustic imaging: Overcoming the physical and practical challenges. Chem. Rev..

[7] Tam A.C. (1986). Applications of photoacoustic sensing techniques. Rev. Mod. Phys..

[8] Park J., Choi S., Knieling F., Clingman B., Bohndiek S., Wang L.V., Kim C. (2024). Clinical translation of photoacoustic imaging. Nat. Rev. Bioeng..

[9] Li C., Wang L.V. (2009). Photoacoustic tomography and sensing in biomedicine. Phys. Med. Biol..

[10] Park B., Oh D., Kim J., Kim C. (2023). Functional photoacoustic imaging: From nano-and micro-to macro-scale. Nano Converg..

[11] Wang L.V. (2009). Multiscale photoacoustic microscopy and computed tomography. Nat. Photonics.

[12] Jeon S., Kim J., Lee D., Baik J.W., Kim C. (2019). Review on practical photoacoustic microscopy. Photoacoustics.

[13] Yao J., Wang L.V. (2014). Sensitivity of photoacoustic microscopy. Photoacoustics.

[14] Wang L.V. (2008). Tutorial on photoacoustic microscopy and computed tomography. IEEE J. Sel. Top. Quantum Electron..

[15] Strohm E.M., Moore M.J., Kolios M.C. (2015). Single cell photoacoustic microscopy: A review. IEEE J. Sel. Top. Quantum Electron..

[16] Yao J., Wang L.V. (2013). Photoacoustic microscopy. Laser Photonics Rev..

[17] Hu S., Maslov K., Wang L.V. (2011). Second-generation optical-resolution photoacoustic microscopy with improved sensitivity and speed. Opt. Lett..

[18] Kim D., Park E., Park J., Perleberg B., Jeon S., Ahn J., Ha M., Kim H.H., Kim J.Y., Jung C.K. (2024). An ultraviolet-transparent ultrasound transducer enables high-resolution label-free photoacoustic histopathology. Laser Photonics Rev..

[19] Cao R., Nelson S.D., Davis S., Liang Y., Luo Y., Zhang Y., Crawford B., Wang L.V. (2023). Label-free intraoperative histology of bone tissue via deep-learning-assisted ultraviolet photoacoustic microscopy. Nat. Biomed. Eng..

[20] Bell K., Abbasi S., Dinakaran D., Taher M., Bigras G., van Landeghem F.K., Mackey J.R., Haji Reza P. (2020). Reflection-mode virtual histology using photoacoustic remote sensing microscopy. Sci. Rep..

[21] Heijblom M., Piras D., Brinkhuis M., van Hespen J.C., van den Engh F.M., van der Schaaf M., Klaase J.M., van Leeuwen T.G., Steenbergen W., Manohar S. (2015). Photoacoustic image patterns of breast carcinoma and comparisons with Magnetic Resonance Imaging and vascular stained histopathology. Sci. Rep..

[22] Martell M.T., Haven N.J., Cikaluk B.D., Restall B.S., McAlister E.A., Mittal R., Adam B.A., Giannakopoulos N., Peiris L., Silverman S. (2023). Deep learning-enabled realistic virtual histology with ultraviolet photoacoustic remote sensing microscopy. Nat. Commun..

[23] Baik J.W., Kim H., Son M., Choi J., Kim K.G., Baek J.H., Park Y.H., An J., Choi H.Y., Ryu S.Y. (2021). Intraoperative label-free photoacoustic histopathology of clinical specimens. Laser Photonics Rev..

[24] Shi J., Wong T.T., He Y., Li L., Zhang R., Yung C.S., Hwang J., Maslov K., Wang L.V. (2019). High-resolution, high-contrast mid-infrared imaging of fresh biological samples with ultraviolet-localized photoacoustic microscopy. Nat. Photonics.

[25] Kim J., Lee J., Choi S., Lee H., Yang J., Jeon H., Sung M., Kim W.J., Kim C. (2024). 3d multiparametric photoacoustic computed tomography of primary and metastatic tumors in living mice. ACS Nano.

[26] Hoelen C., De Mul F., Pongers R., Dekker A. (1998). Three-dimensional photoacoustic imaging of blood vessels in tissue. Opt. Lett..

[27] Kolkman R.G., Klaessens J.H., Hondebrink E., Hopman J.C., de Mul F.F., Steenbergen W., Thijssen J.M., van Leeuwen T.G. (2004). Photoacoustic determination of blood vessel diameter. Phys. Med. Biol..

[28] Toi M., Asao Y., Matsumoto Y., Sekiguchi H., Yoshikawa A., Takada M., Kataoka M., Endo T., Kawaguchi-Sakita N., Kawashima M. (2017). Visualization of tumor-related blood vessels in human breast by photoacoustic imaging system with a hemispherical detector array. Sci. Rep..

[29] Li M., Tang Y., Yao J. (2018). Photoacoustic tomography of blood oxygenation: A mini review. Photoacoustics.

[30] Hu S., Wang L.V. (2010). Photoacoustic imaging and characterization of the microvasculature. J. Biomed. Opt..

[31] Ahn J., Kim J.Y., Choi W., Kim C. (2021). High-resolution functional photoacoustic monitoring of vascular dynamics in human fingers. Photoacoustics.

[32] Rich L.J., Seshadri M. (2015). Photoacoustic imaging of vascular hemodynamics: Validation with blood oxygenation level–dependent MR imaging. Radiology.

[33] Zhu X., Huang Q., DiSpirito A., Vu T., Rong Q., Peng X., Sheng H., Shen X., Zhou Q., Jiang L. (2022). Real-time whole-brain imaging of hemodynamics and oxygenation at micro-vessel resolution with ultrafast wide-field photoacoustic microscopy. Light Sci. Appl..

[34] Kim J., Choi S., Kim C., Kim J., Park B. (2024). Review on Photoacoustic Monitoring after Drug Delivery: From Label-Free Biomarkers to Pharmacokinetics Agents. Pharmaceutics.

[35] Zhang Y., Yu J., Kahkoska A.R., Gu Z. (2017). Photoacoustic drug delivery. Sensors.

[36] Xia J., Kim C., Lovell J.F. (2015). Opportunities for photoacoustic-guided drug delivery. Curr. Drug Targets.

[37] Moore C., Jokerst J.V. (2019). Strategies for image-guided therapy, surgery, and drug delivery using photoacoustic imaging. Theranostics.

[38] Feng Q., Zhang Y., Zhang W., Shan X., Yuan Y., Zhang H., Hou L., Zhang Z. (2016). Tumor-targeted and multi-stimuli responsive drug delivery system for near-infrared light induced chemo-phototherapy and photoacoustic tomography. Acta Biomater..

[39] Manivasagan P., Bharathiraja S., Bui N.Q., Jang B., Oh Y.-O., Lim I.G., Oh J. (2016). Doxorubicin-loaded fucoidan capped gold nanoparticles for drug delivery and photoacoustic imaging. Int. J. Biol. Macromol..

[40] Park B., Park S., Kim J., Kim C. (2022). Listening to drug delivery and responses via photoacoustic imaging. Adv. Drug Deliv. Rev..

[41] Maslov K., Zhang H.F., Hu S., Wang L.V. (2008). Optical-resolution photoacoustic microscopy for in vivo imaging of single capillaries. Opt. Lett..

[42] Hu S., Wang L.V. (2013). Optical-resolution photoacoustic microscopy: Auscultation of biological systems at the cellular level. Biophys. J..

[43] Bi R., Ma Q., Mo H., Olivo M., Pu Y. (2019). Optical-resolution photoacoustic microscopy of brain vascular imaging in small animal tumor model using nanosecond solid-state laser. Neurophotonics and Biomedical Spectroscopy.

[44] Kim J.Y., Lee C., Park K., Lim G., Kim C. (2015). Fast optical-resolution photoacoustic microscopy using a 2-axis water-proofing MEMS scanner. Sci. Rep..

[45] Cho S.-W., Park S.M., Park B., Lee T.G., Kim B.-M., Kim C., Kim J., Lee S.-W., Kim C.-S. (2021). High-speed photoacoustic microscopy: A review dedicated on light sources. Photoacoustics.

[46] Wong T.T., Zhang R., Hai P., Zhang C., Pleitez M.A., Aft R.L., Novack D.V., Wang L.V. (2017). Fast label-free multilayered histology-like imaging of human breast cancer by photoacoustic microscopy. Sci. Adv..

[47] Liu W., Shcherbakova D.M., Kurupassery N., Li Y., Zhou Q., Verkhusha V.V., Yao J. (2018). Quad-mode functional and molecular photoacoustic microscopy. Sci. Rep..

[48] Cao R., Zhao J., Li L., Du L., Zhang Y., Luo Y., Jiang L., Davis S., Zhou Q., de la Zerda A. (2023). Optical-resolution photoacoustic microscopy with a needle-shaped beam. Nat. Photonics.

[49] Park J., Park B., Kim T.Y., Jung S., Choi W.J., Ahn J., Yoon D.H., Kim J., Jeon S., Lee D. (2021). Quadruple ultrasound, photoacoustic, optical coherence, and fluorescence fusion imaging with a transparent ultrasound transducer. Proc. Natl. Acad. Sci. USA.

[50] Cho S., Kim M., Ahn J., Kim Y., Lim J., Park J., Kim H.H., Kim W.J., Kim C. (2024). An ultrasensitive and broadband transparent ultrasound transducer for ultrasound and photoacoustic imaging in-vivo. Nat. Commun..

[51] Chen H., Agrawal S., Dangi A., Wible C., Osman M., Abune L., Jia H., Rossi R., Wang Y., Kothapalli S.-R. (2019). Optical-resolution photoacoustic microscopy using transparent ultrasound transducer. Sensors.

[52] Park J., Park B., Ahn J., Kim D., Kim J.Y., Kim H.H., Kim C. (2022). Opto-ultrasound biosensor for wearable and mobile devices: Realization with a transparent ultrasound transducer. Biomed. Opt. Express.

[53] Kim J., Heo D., Cho S., Ha M., Park J., Ahn J., Kim M., Kim D., Jung D.H., Kim H.H. (2024). Enhanced dual-mode imaging: Superior photoacoustic and ultrasound endoscopy in live pigs using a transparent ultrasound transducer. Sci. Adv..

[54] Park J., Ahn J., Ban S., Park E., Lee H., Choi T., Kim C. (2024). Multicontrast and Nondestructive Transparent Ultrasound Transducer Based Photoacoustic and Optical Coherence Imaging of Multilayered Electronics. IEEE Trans. Instrum. Meas..

[55] Park J., Park B., Yong U., Ahn J., Kim J.Y., Kim H.H., Jang J., Kim C. (2022). Bi-modal near-infrared fluorescence and ultrasound imaging via a transparent ultrasound transducer for sentinel lymph node localization. Opt. Lett..

[56] Yao J., Huang C.-H., Wang L., Yang J.-M., Gao L., Maslov K.I., Zou J., Wang L.V. (2012). Wide-field fast-scanning photoacoustic microscopy based on a water-immersible MEMS scanning mirror. J. Biomed. Opt..

[57] Kim J., Kim J.Y., Jeon S., Baik J.W., Cho S.H., Kim C. (2019). Super-resolution localization photoacoustic microscopy using intrinsic red blood cells as contrast absorbers. Light Sci. Appl..

[58] Liao T., Liu Y., Wu J., Deng L., Deng Y., Zeng L., Ji X. (2021). Centimeter-scale wide-field-of-view laser-scanning photoacoustic microscopy for subcutaneous microvasculature in vivo. Biomed. Opt. Express.

[59] Zafar M., McGuire L.S., Ranjbaran S.M., Matchynski J.I., Manwar R., Conti A.C., Perrine S.A., Avanaki K. (2024). Spiral laser scanning photoacoustic microscopy for functional brain imaging in rats. Neurophotonics.

[60] Zafar M., Manwar R., McGuire L.S., Charbel F.T., Avanaki K. (2023). Ultra-widefield and high-speed spiral laser scanning OR-PAM: System development and characterization. J. Biophotonics.

[61] Lan B., Liu W., Wang Y.-c., Shi J., Li Y., Xu S., Sheng H., Zhou Q., Zou J., Hoffmann U. (2018). High-speed widefield photoacoustic microscopy of small-animal hemodynamics. Biomed. Opt. Express.

[62] Cho S., Baik J., Managuli R., Kim C. (2020). 3D PHOVIS: 3D photoacoustic visualization studio. Photoacoustics.

[63] McLean G. (1988). Review of recent progress in linear motors. IEE Proc. B (Electr. Power Appl.).

[64] Choi S., Kim J.Y., Lim H.G., Baik J.W., Kim H.H., Kim C. (2020). Versatile single-element ultrasound imaging platform using a water-proofed MEMS scanner for animals and humans. Sci. Rep..

[65] Park E.-Y., Park S., Lee H., Kang M., Kim C., Kim J. (2021). Simultaneous dual-modal multispectral photoacoustic and ultrasound macroscopy for three-dimensional whole-body imaging of small animals. Photonics.

[66] Kim J., Park B., Ha J., Steinberg I., Hooper S.M., Jeong C., Park E.-Y., Choi W., Liang T., Bae J.S. (2021). Multiparametric photoacoustic analysis of human thyroid cancers in vivo. Cancer Res..

[67] Zhang E., Laufer J., Beard P. (2008). Backward-mode multiwavelength photoacoustic scanner using a planar Fabry-Perot polymer film ultrasound sensor for high-resolution three-dimensional imaging of biological tissues. Appl. Opt..

[68] Hajireza P., Forbrich A., Zemp R. (2014). In-vivo functional optical-resolution photoacoustic microscopy with stimulated Raman scattering fiber-laser source. Biomed. Opt. Express.

[69] Lee H., Park S.M., Park J., Cho S.-W., Han S., Ahn J., Cho S., Kim C., Kim C.-S., Kim J. (2024). Transportable Multispectral Optical-Resolution Photoacoustic Microscopy using Stimulated Raman Scattering Spectrum. IEEE Trans. Instrum. Meas..

[70] Choi S., Yang J., Lee S.Y., Kim J., Lee J., Kim W.J., Lee S., Kim C. (2023). Deep learning enhances multiparametric dynamic volumetric photoacoustic computed tomography in vivo (DL-PACT). Adv. Sci..

[71] Zhang H.F., Maslov K., Sivaramakrishnan M., Stoica G., Wang L.V. (2007). Imaging of hemoglobin oxygen saturation variations in single vessels in vivo using photoacoustic microscopy. Appl. Phys. Lett..

[72] Glatz J., Deliolanis N.C., Buehler A., Razansky D., Ntziachristos V. (2011). Blind source unmixing in multi-spectral optoacoustic tomography. Opt. Express.

